# Correction to: Chest x-ray severity score in COVID-19 patients on emergency department admission: a two-centre study

**DOI:** 10.1186/s41747-021-00215-3

**Published:** 2021-04-14

**Authors:** Cristian Giuseppe Monaco, Federico Zaottini, Simone Schiaffino, Alessandro Villa, Gianmarco Della Pepa, Luca Alessandro Carbonaro, Laura Menicagli, Andrea Cozzi, Serena Carriero, Francesco Arpaia, Giovanni Di Leo, Davide Astengo, Ilan Rosenberg, Francesco Sardanelli

**Affiliations:** 1 Unit of Radiology, IRCCS Policlinico San Donato, Via Rodolfo Morandi 30, 20097 San Donato Milanese, Italy; 2 Unit of Radiology, Ospedale Lavagna, Via Don Giovanni Battista Bobbio 25, 16033 Lavagna, Italy; 3 Postgraduate School in Radiodiagnostics, Università degli Studi di Milano, Via Festa del Perdono 7, 20122 Milan, Italy; 4 Department of Biomedical Sciences for Health, Università degli Studi di Milano, Via Luigi Mangiagalli 31, 20133 Milan, Italy

**Correction to: Eur Radiol Exp 4, 68 (2020)**

**https://doi.org/10.1186/s41747-020-00195-w**

The original article [1] mistakenly omitted a declaration of competing interests. An appropriate statement can be viewed ahead:

**Competing Interests**

Francesco Sardanelli is the Editor-in-Chief of European Radiology Experimental; for this reason, he was not involved in any way in the revision/decision process, which was completely managed by the Guest Editor, Prof. Akos Varga-Szemes (Medical University of South Carolina, Charleston, SC, USA).
